# Investigation of stress in dogs during an MRI examination in response to hearing protection

**DOI:** 10.1371/journal.pone.0312166

**Published:** 2024-10-17

**Authors:** Maike Schroers, Y. Zablotski, Andrea Meyer-Lindenberg

**Affiliations:** Veterinary Faculty, Clinic of Small Aninmal Surgery and Reproduction, Ludwig-Maximilians-Universität München, München, Germany; University of Lincoln - Brayford Campus: University of Lincoln, UNITED KINGDOM OF GREAT BRITAIN AND NORTHERN IRELAND

## Abstract

The aim of the present study was to investigate whether the wearing of hearing protection has a positive influence on stress levels during an MRI examination in dogs under anaesthesia. To this end, the stress hormones cortisol and arginine vasopressin (AVP) were measured in the saliva of patients wearing hearing protection during an MRI scan, as well as in the control group without hearing protection, before and after the scan. Pulse rate and noise level were also measured during the MRI. It was shown that salivary cortisol concentrations in dogs without hearing protection increased during the MRI examination, while those in the control group with hearing protection remained the same (p<0.05). The pulse range was greater in the control group without hearing protection, although not statistically significant. The maximum loudness during the MRI examination, measured at 200 cm from the gantry, was 87 dB(A). The results on the loudness during mri scan highlight that hearing protection should always be used to minimise hearing damage and possibly the stress level for dogs.

## Introduction

In small animal medicine, magnetic resonance imaging (MRI) is a common modality of diagnostic imaging [1–5]. In general, animals, unlike humans, undergo anaesthesia during the examination. In human medicine, the patient receives hearing protection during the examination due to the considerable noise level.

The noise level during an MRI examination can vary, depending on the device and the sequences [6]. However, in an experimental study comparing the noise intensity of MRIs with the hearing threshold of monkeys, dogs, cats, pigs, and rabbits, the authors generally advocated the use of hearing protection in all animal species during an MRI examination [7]. In another clinical study, distortion product otoacoustic emission (DPOAE) was used to assess cochlear function in dogs before and after MRI, demonstrating reduced cochlear function five minutes after MRI [8]. With regard to long-term damage, more extensive studies are still lacking.

A clinical study in awake dogs showed that auditory stimulation, such as the sounds of a wet/dry vacuum cleaner, did not increase the serum stress hormone cortisol in dogs [9]. The effect of noise on cortisol and arginine vasopressin (AVP) concentrations in saliva of dogs was investigated in another clinical study. The animals were exposed to a defined noise level for 30 min using a vacuum device, which led to changes in cortisol and AVP concentrations in saliva [10]. No study is yet available in veterinary medicine on auditory stimulation of stress levels in patients under anesthesia.

Cortisol represents a hormone which is regulated by the hypothalamic-pituitary-adrenal (HPA) axis [11,12]. In both human and veterinary medicine, it represents a well-known parameter associated with physical and psychological stress and pain [13–15].

AVP, which has also been studied as a parameter for assessing stress, is a peptide hormone produced in the hypothalamus, stored in the pituitary gland, and released when needed [16]. The principal role of ADH is to increase water reabsorption in the kidneys. The most well-known function is thus the antidiuretic effect in water balance, which is why AVP is also called anti-diuretic hormone (ADH). Like cortisol, it has an influence on the contraction of blood vessels and thus on blood pressure [17]. Likewise, AVP is used in both human [18–20] and animal [21,22] clinical trials to assess stress and pain.

Various parameters of stress such as cortisol, AVP or pulse rate may be influenced by arousal which can be influenced by positive or negative emotions [23].

Since there have been no studies to date on stress in dogs during MRI examinations, the purpose of the present study was to investigate whether wearing hearing protection during an MRI examination in dogs under anaesthesia leads to a reduced stress response in the patient. Even though the dogs are under anaesthesia, the ear as a sensory organ is constantly exposed to acoustic irradiation during the examination, so stress and damage to the hearing may be possible during the examination. It should be investigated whether a resulting stress response could be demonstrated by cortisol and AVP concentrations in the saliva of dogs with and without hearing protection. In additional, it should be verified whether a change in pulse rate during anesthesia monitoring can provide additional evidence of stress reactions during an MRI examination.

## Materials and methods

The present study included dogs that underwent MRI because of a suspected cruciate ligament tear in the stifle joint. The dogs referred to were patients of the Surgical and Gynecological Small Animal Clinic, Ludwig-Maximilians-University Munich. Breed, sex, age and weight of the patients were recorded as part of the medical history. Patients with an injury to the oral cavity, in whom saliva sampling was therefore not possible, were excluded. Patients with previously diagnosed diseases of the brain or adrenal gland as well as patients with obesity or other painful diseases were also excluded, as these factors may influence the release of cortisol and AVP. Furthermore, patients with pre-reporting diseases of the ear (e.g. otitis) were excluded, as these may affect the hearing ability of the dogs. As this was a pilot study, the authors decided on a group size of at least 15 animals per group, based on the recommendations from the literature [24,25]. The studies were approved by the Ethics Committee of the Faculty of Veterinary Medicine, Ludwig-Maximilians-University, Munich (reference number 271-14-06-2021).

**Fig 1:**
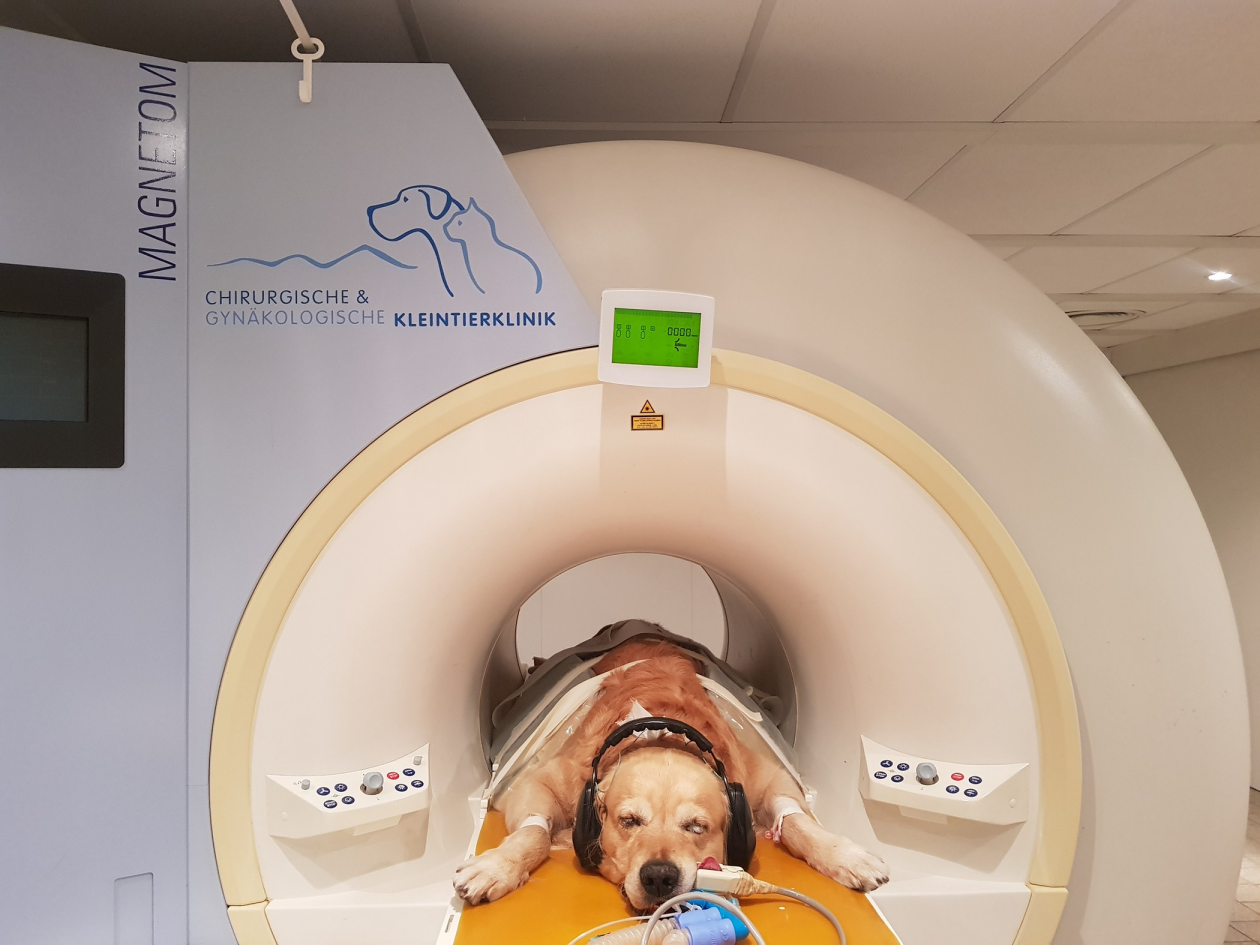
Patient in the study group wearing hearing protection before an MRI examination.

**Fig 2:**
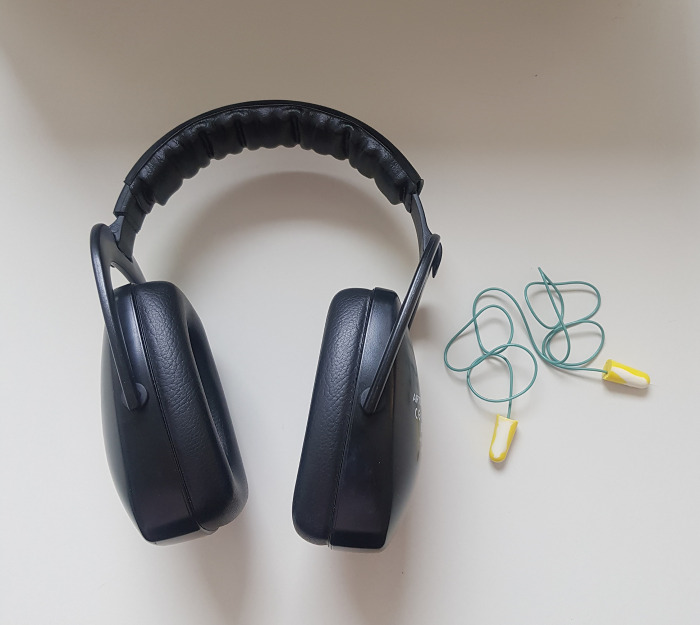
Headphones (MRI earmuffs, allMRI GmbH, Nordheim, Germany) and earplugs (BILSOM ®304S EARPLUG, Honeywell, Germany) with safety cord.

During the MRI examination under anesthesia, the patients in the study group received earplugs (BILSOM ®304S EARPLUG, Honeywell, Germany) and hearing protection (MRI earmuffs, allMRI GmbH, Nordheim, Germany) covering the auricles (Figs 1 and 2). Overall, care was taken to ensure that the earplugs sealed the complete auditory canal and that the headphones completely covered the pinnae. The control group received no hearing protection. Dogs were randomly assigned to the study or control group by drawing lots.

Dogs were given ad libitum access to water prior to anaesthesia. None of the dogs were dehydrated on clinical examination prior to induction. The dogs were sedated with Diazepam (Solupam, Dechra, Germany) (0.5 mg/kg i.v.) and propofol (Narcofol, CP-Pharma, Germany) (2–4 mg/kg i.v.) as part of the regular induction of anesthesia before being intubated. Subsequently, anesthesia was maintained during the MRI examination with inhalation anesthesia (isoflurane 1.2–1.4% by volume). In addition, patients received a crystalloid infusion (5 ml/kg/h) (Elektrosel, selectavet, Germany) and were constantly monitored by an anesthesiologist.

In addition to respiratory parameters such as oxygen saturation and end-expiratory CO2 pulse rate was documented using a pulse oximeter on the tongue (interval every 5 min) as part of the general anesthetic monitoring. The pulse rate was also used to determine the pulse range in order to investigate how strongly it fluctuated during the MRI scan. All patients were positioned in the thoracic recumbent position. MRI was acquired of the knees according to a standardized protocol (Table 1).

**Table 1:** Standardized MRI protocol for knee joint examination for the examination and control groups of the present study (total duration 32 min).

Sequence	Time (min)
lokalizer	01:03
transversal T1 3mm 320	03:15
dorsal PD tse fs 384	05:25
sagital PD tse fs 384	07:17
transversal PD fs 3mm	10:14
sagital T2 tse rst 2,5 mm	04:36

PD: Proton density; fs: Fat suppression, tse = turbo spin echo.

Saliva samples were taken from all patients at the same time according to a standardised protocol before and after the MRI scan to measure the stress hormones cortisol and AVP. The time between induction of anaesthesia and first saliva collection prior to MRI was estimated to be ten minutes. The second sample, also taken while the patient was under anaesthetic, was taken immediately after the MRI. For saliva collection, a salivette (Children Swab, Salimetrics® LLC, USA) was placed in the patient’s mouth (under the tongue and in the lateral cheek pocket) for one minute to collect saliva. The saliva was then stored in a suitable tube (Storage Tubes, Salimetrics® LLC, USA), centrifuged, and the saliva transferred with a pipette to a microcentrifuge tube (Eppendorf tube) and frozen (-20°C) until measurement. The determination of AVP in the saliva samples (minimum 10 μl/sample) was performed in the in-house laboratory using commercially available enzyme-linked immunosorbent assay (ELISA) kits (arginine vasopressin ELISA kit, Enzo Liefe Science®, Lörrach, Germany). For the determination of cortisol, saliva samples (at least 50μl/sample) were sent refrigerated to the Faculty of Psychology at the Technical University of Dresden, Germany, where they were measured and analysed by chemiluminescence immunoassay (CLIA) (IBL International, Hamburg, Germany).

During the MRI examination, noise intensity was measured using a sound level measurement application (App, Sound Meter, Tools Dev) on a smartphone (Galaxy 8, Samsung Electronics Co., Ltd.) placed 200 cm from the gantry.

### Statistical analysis

The data for age, bodyweight, pulse range, average pulse, pulse minimum and pulse maximum were first checked die for normality via the Shapiro-Wilk test. In case data was not normally distributed (pulse range, age and bodyweight), Mann-Whitney test was applied to compare two groups (control and ear protection). In case they were normally distributed, the Levene test was applied to access for homogeneity of variance across groups. Since three remmaining parameters (average pulse, pulse minimum and pulse maximum) showed equal variances, the Student’s t-test was employed to compare two groups (control and ear protection).

The data for vasopressin and cortisol were dependend, where two timepoints, pre mri and post mri, supposed to be compared. Since the data for both vasopressin and cortisol was not-normally distributed, we modeled the logarithm of them via the mixed effects linear models. The residuals of models were normally distributed and no evidence for different variances across groups (Bartlett Test, p = 0.531) was found.

## Results

A total of 73 patients were included in the study, including 31 study animals wearing hearing protection during the MRI examination and 42 patients in the control group without hearing protection. The mean age was 6 years (range 1–11) and a mean weight of 30 kg (range 9–73). Statistical analysis showed no differences in age or weight between the examination and control groups.

**Fig 3:**
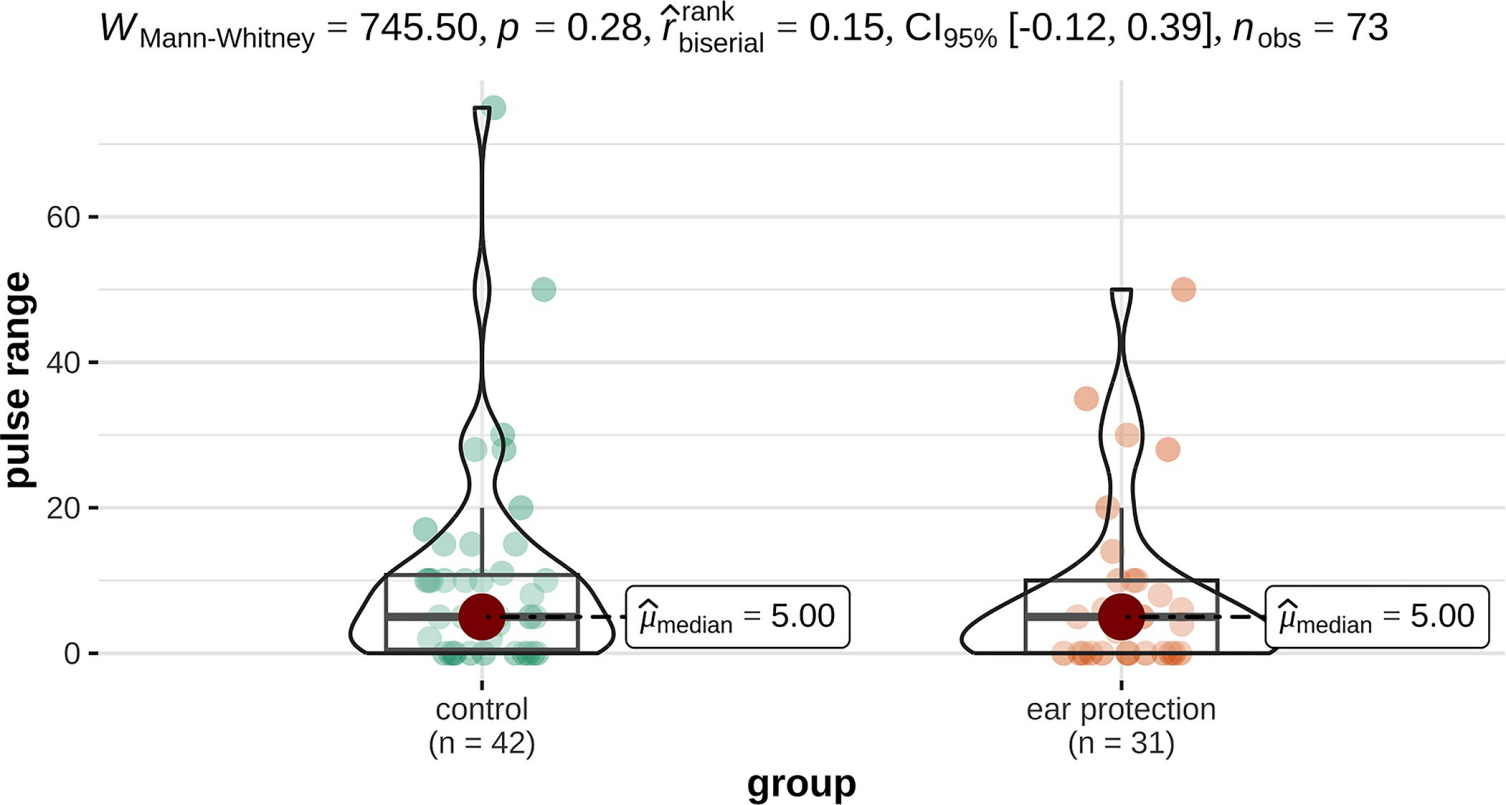
Log vasopressin concentrations in saliva before and after MRI in the study group (n = 16) with hearing protection and in the control group (n = 16).

The mean pulse rate in the study group (n = 31) was 105 bpm, and the pulse range during the MRI scan was 5bpm. In the control group (n = 42), the mean pulse rate was 110 bpm and the pulse range was 12 bpm. Thus, the study group with hearing protection tended to have a lower pulse range even though the difference was not statistically significant (p = 0.06). There were also no differences in the maximum and mean pulse rates between the study and control groups (p = 0.28) (Fig 3).

**Fig 4:**
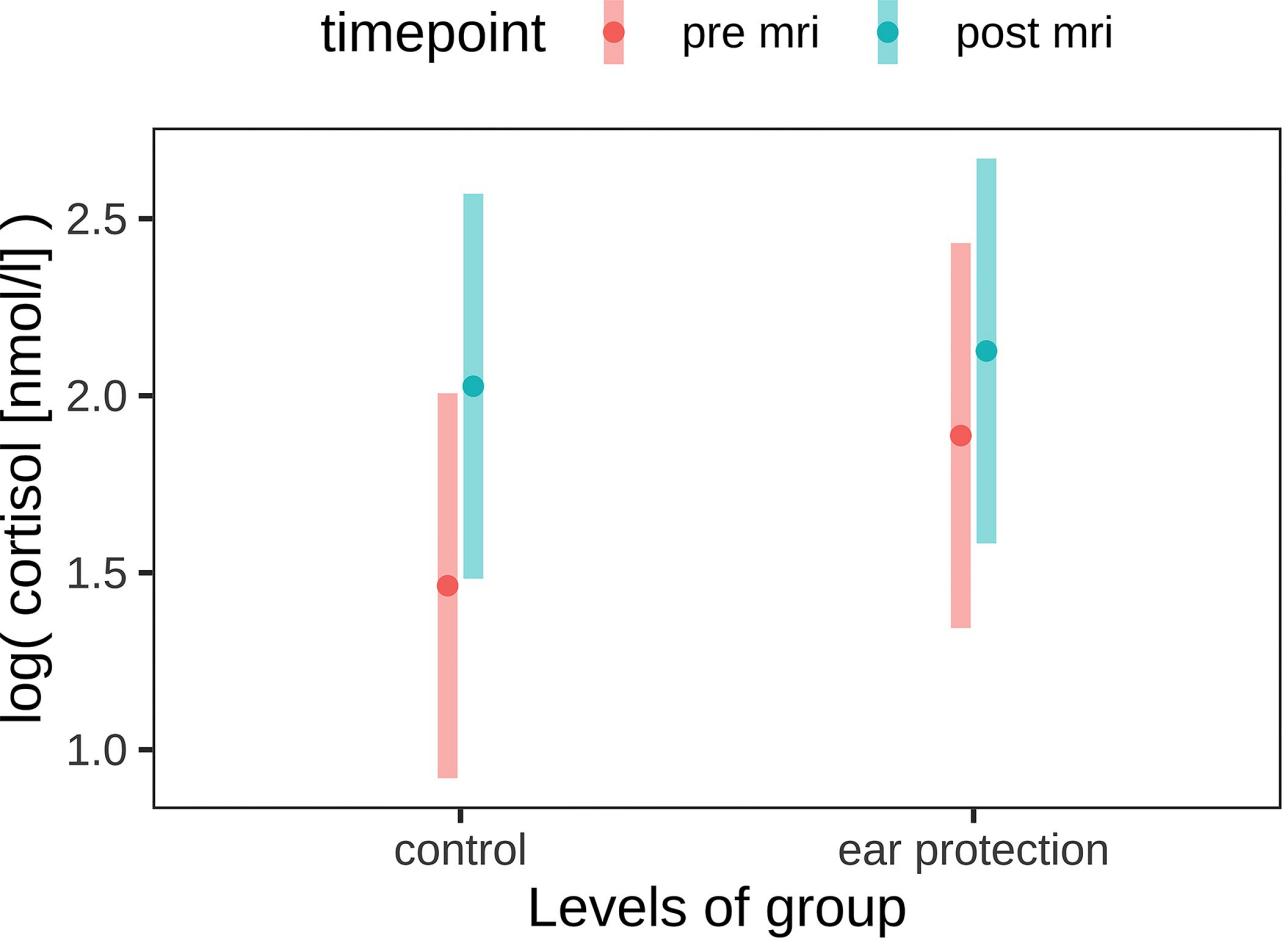
Range of pulse rate (bpm) of examination (right) and control group (left) during MRI examination.

Due to the limited amount of saliva obtained, it was not possible to determine both stress hormones in all patients. Salivary cortisol concentrations were measured in 30/73 patients (study group n = 15, control group n = 15). The mean age in this group was 5 years in the study group (range 1–11) and 7 years (1–11) in the control group. Mean weight was 33kg in the study group (range 20–48) and 27kg in the control group (range 10–39). It was evident that the cortisol concentrations in the dogs of the control group without hearing protection increased during the MRI examination (p = 0.05), whereas they remained the same in the study group with hearing protection (p = 0.39) (Fig 4).

**Fig 5:**
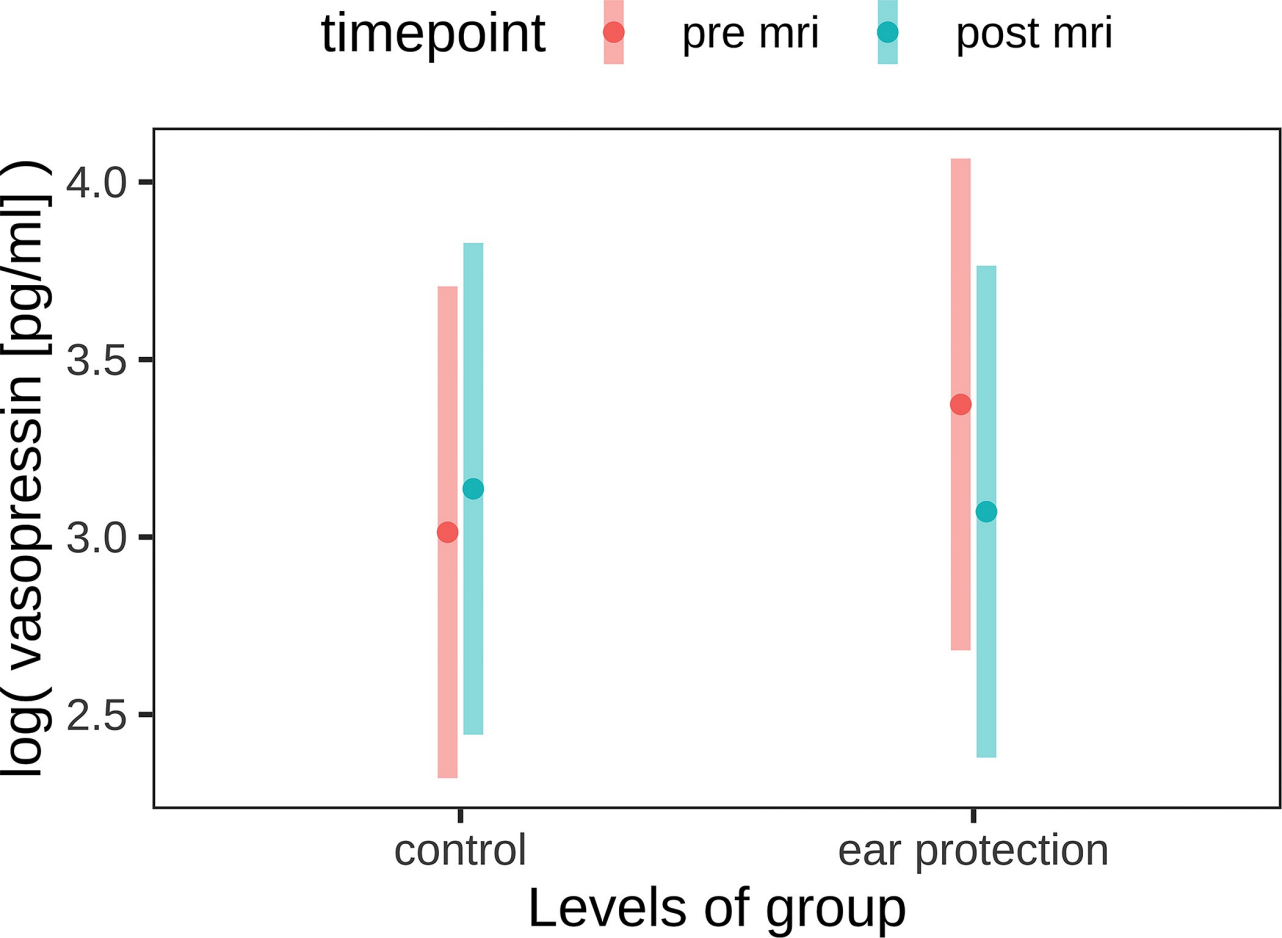
Logarithmized salivary cortisol concentrations before and after MRI in the study group (n = 15) with hearing protection and in the control group (n = 15).

AVP concentrations in saliva were measured in 32/73 patients (study group n = 16; control group n = 16). Here, the mean age of the study group was 5 years (range 3–11), that of the control group was 6 years (range 1–11). With regard to salivary AVP concentrations, these decreased in the study group, although the decrease was not statistically significant (p = 0.34); in the control group, no difference was found before and after MRI either (Fig 5). The mean pulse rate was 110 bpm in the study group and 109 bpm in the control group. The mean pulse range was significantly lower in the study group (6 bpm) than in the control group (10 bpm); the difference was not statistically significant (p = 0.44).

The length of the MRI scan was 32 min in all patients; the maximum noise level 200 cm away from the gantry was 87dB(A) in both groups, and the average noise level was 76 dB(A).

## Discussion

The aim of the present study was to investigate whether parameters such as pulse rate and salivary cortisol or AVP concentrations provide an indication of stress during an MRI examination in dogs with and without hearing protection. Differences in salivary cortisol fluctuation may indicate a difference in stress in response to hearing protection. Based on the noise levels measured during the MRI examination in the present study and the salivary cortisol concentrations measured in the dogs, it can be concluded that hearing protection should always be used during an MRI examination.

A clinical study showed that auditory stimulation, such as the sounds of a wet/dry vacuum cleaner, in awake dogs did not result in a stress response and increased serum cortisol concentrations in the animals studied [9]. This highlights the challenge that a potential stress response to an auditory stimulus may not necessarily lead to an increase in salivary cortisol. In the present study, salivary cortisol levels increased in the control group without hearing protection, possibly due to stress caused by the noise level during the MRI scan. In patients with hearing protection, cortisol levels remained the same (p>0.05), possibly preventing a similar stress response due to noise exposure during the scan.

No increase or decrease in salivary AVP concentrations was observed during the MRI study in either the study or control groups. In a clinical study of separation pain in dogs, it was shown that within three minutes of a stressful situation, increased salivary AVP concentrations could be detected [21]. A limitation of the present study is that saliva samples were taken only before and after the MRI examination. Therefore, the exact course of AVP concentrations over the course of the study could not be assessed and possible increases or decreases may have been missed.

With regard to anaesthesia and its monitoring, the use of pulse rate under anaesthesia to assess a patient’s stress response is limite [26]. Thus, although the pulse range of patients without hearing protection was greater than that of the study group, the difference was not statistically significant. Studies with larger numbers of patients would be useful to investigate whether stress reduction with hearing protection is associated with reduced pulse variability.

The measured noise level of the MRI examination at 200 cm from the gantry was 76 dB(A) in both the examination and control groups of the present study, and the maximum noise level was 87 dB(A). These results are also in line with a study by Price et al (2001) in which the sound levels of 15 MRI scanners were comparable and ranged from 82.5 to 118.4 dB(A). The measurement in the present study was made using the ’Sound meter dB’ app and recorded on a smartphone placed approximately 200 cm from the MRI scanner. The measurement was only a rough estimate of the acoustic loudness, as it was technically impossible to measure the loudness directly at the patient’s ear in the gantry. Due to the distance of the device from the patient’s ear, it is likely that the measured volumes were lower than the actual volume measured at the ear. Therefore, Lauer et al. (2012) compared the loudness measured at the patient’s ear (97.3 dB(A)) with the loudness measured at a distance of 157 cm from the isocentre and thus outside the gantry (92.7 dB(A)). Approximately 200 cm away, the loudness was 88 dB(A) [7], similar to the present study.

In a study by Gin et al. (2018), awake dogs were exposed to a wet/dry vacuum cleaner for 30 minutes at noise levels of 85–95 dB(A) (9). In another study in dogs, the parameter IgA was investigated as a marker of stress. It was shown that a loudness of 75–78 dB(A) was associated with an increase in salivary IgA concentrations in the awake study group [27]. Thus, it is possible that the loudness of the MRI examinations in the present study induced a stress response in the group of dogs without hearing protection and led to an increase in salivary cortisol concentrations, whereas the concentrations in the group of dogs with hearing protection remained unchanged. An MRI-compatible sound level meter (Hottinger Brüel& Kjaer GmbH, Darmstadt, Germany), for example, is suitable for accurate measurement directly on the patient. The main difference with previous noise exposure studies is that the animals were under anaesthesia during the study, and there is no comparable literature to date.

There were no known hearing problems and the animals responded normally. However, negative effects of noise intensity on the auditory organs could not be investigated in this study. An experimental study was conducted to assess the risk of possible damage to the inner ear, and thus hearing, in experimental and domestic animals during MRI examinations [7]. The authors compared MRI noise levels with the hearing thresholds of monkeys, dogs, cats, pigs and rabbits. Using the US National Institute for Occupational Safety and Health (NIOSH) recommendations for safe noise levels, the authors advocate the use of hearing protection during MRI in all species [7]. Furthermore, in the study by Venn et al. (2014), a short-term loss of cochlear function was observed in dogs immediately after a single MRI. As the loudness of the MRI was comparable to that of the other studies and the long-term effects are unknown, the authors of the present study also advocate hearing protection for single MRIs.

It is difficult to determine the specific noise level at which non-reversible damage to the inner ear may occur after a single exposure under anaesthesia. It depends on the species and other factors such as the age of the animals. For example, the hearing organs of younger animals are more sensitive than those of older animals [28]. The age of the dogs in the present study was 6 years (range 1–11), so that neither very young nor very old animals were excluded. It should also be borne in mind that natural protective responses to noise, such as the inner ear reflex, may be absent during anaesthesia [29,30]. Concrete studies of damage to the inner ear and the risk of tinnitus from auditory stimulation have only been carried out on mice exposed to a noise level of 100 dB for two hours, resulting in damage to the inner ear [31]. However, these are difficult to compare with the present study because the MRI scans in the present study were shorter (32 minutes) and the inner ear was not exposed to a continuous volume level, but only to intermittent volume pulses. To minimise the risk of damage to the inner ear, the use of hearing protection is always recommended.

Ear protection for the study group consisted of earplugs and headphones. Although care was taken to ensure that the earplugs sealed the entire ear canal and the headphones completely covered the auricle, the extent to which this protection blocked all noise was not assessed. Experimental hearing tests would be needed to determine how well the earplugs and hearing protectors blocked out sound, which may depend on the anatomy of the ear canal.

The timing of saliva collection was standardised in this study. Therefore, saliva samples for cortisol and AVP were collected immediately before and after the MRI scan. Approximately ten minutes elapsed between induction of the awake patient with propofol sedation and the MRI scan. In general, it can be assumed that the psychological stress of the animals in the veterinary clinic also influenced the results. For example, several clinical studies have shown that dogs in veterinary clinics have significantly increased stress level [32–34]. Due to preoperative anxiety, it could be assumed that the stress level before the MRI examination could be significantly higher than after the examination. A prolonged period between induction of anaesthesia and saliva collection was avoided for ethical reasons to minimise the duration of anaesthesia.

Cruciate ligament rupture itself is always associated with inflammation in the knee joint, which also affects the release of stress hormones in the body. In order to keep the protocol as consistent as possible, only patients with the same disease and the same MRI protocol were included in the experimental and control groups. In addition, only progression, i.e. an increase or decrease in hormone concentrations, was assessed during the MRI scan. As the patients were in a well-fixed position under anaesthesia, it can be assumed that the pain caused by the inflammation in the knee joint did not lead to significant fluctuations in hormone concentrations or pulse rate during the MRI scan.

Blood pressure could not be measured in this study because of the equipment available. This is a limitation of the study, as cortisol and AVP can affect blood pressure. However, all patients received a continuous crystalloid infusion (5 ml/kg/h) to minimise the risk of hypotension. It should be noted that a significant drop in blood pressure could also affect pulse rate, but no differences were found between the study and control groups.

The extent to which inhalational anaesthesia with isoflurane has an effect on salivary cortisol concentrations was not investigated in the present study. There is no evidence in the available literature that diazepam, propofol or isoflurane affect the auditory threshold. Therefore, these two drugs were used in the standardised anaesthetic protocol for both the study and control groups.

## Conclusion

In conclusion, hearing protection during canine MRI is indicated to minimise hearing damage and possibly stress to the patient. Earplugs and headphones are therefore cost-effective options for canine hearing protection that should be used in any clinic where MRI is performed.
